# Leucyl-tRNA synthetase as a molecular target of isobutanol-mediated growth inhibition in *Saccharomyces cerevisiae*

**DOI:** 10.1016/j.jbc.2026.113228

**Published:** 2026-06-04

**Authors:** Mano Hasegawa, Nodoka Oshimura, Kaho Hitomi, Ayako Furukawa, Kenji Sugase, Kouichi Kuroda

**Affiliations:** 1 Division of Applied Life Sciences, Graduate School of Agriculture, Kyoto University, Kyoto, Japan; 2 Faculty of Agriculture, Department of Applied Life Sciences, Kyoto University, Kyoto, Japan; 3 Faculty of Molecular Chemistry and Engineering, Graduate School of Science and Technology, Kyoto Institute of Technology, Kyoto, Japan

**Keywords:** *Saccharomyces cerevisiae*, isobutanol, leucyl-tRNA synthetase, BCAAs, competitive inhibition, NMR

## Abstract

Our dependence on fossil fuels and ongoing environmental degradation urgently require sustainable energy solutions. Biofuels that do not rely on fossil resources have attracted significant scientific interest. Among these, isobutanol produced by yeast is a promising next-generation biofuel owing to its high-energy density and compatibility with existing fuel infrastructure. However, the inherently low tolerance of yeast to isobutanol constrains its production, posing a major barrier to the large-scale implementation of bio-based isobutanol. Here, we provide compelling evidence that supplementation with excess branched-chain amino acids (BCAAs), particularly leucine, alleviates isobutanol-induced growth inhibition in yeast. Using saturation transfer difference nuclear magnetic resonance (STD-NMR) spectroscopy and structural modeling, we showed that isobutanol directly interacts with cytoplasmic leucyl-tRNA synthetase (LeuRS), acting as a competitive inhibitor of leucine binding. Collectively, these findings reveal a mechanistic basis by which isobutanol inhibits yeast growth, offering new insights into enhancing isobutanol tolerance and production in yeast.

Amid rising environmental challenges and a persistent reliance on fossil fuels, efforts to develop sustainable energy alternatives have intensified (1). One promising approach to mitigating these issues involves developing biofuels derived from renewable resources. These fuels have gained attention as carbon-neutral energy sources that can serve as alternatives to fossil fuels and are considered essential for achieving a sustainable society (2, 3). Among these, bioethanol is the most widely established biofuel and has already been produced on a commercial scale. However, the physicochemical properties of ethanol—including its high hygroscopicity, volatility, and corrosiveness—limit its range of applications, underscoring the need for alternative biofuels with improved fuel properties (4).

Isobutanol has attracted attention as a promising next-generation biofuel and, more recently, as a feedstock for versatile chemical applications (5, 6, 7, 8). Compared with ethanol, isobutanol exhibits a higher energy density, lower hygroscopicity and corrosiveness, and reduced volatility, which collectively enhance its compatibility with existing fuel infrastructure (5, 6, 7), positioning it as a viable gasoline alternative and a potential candidate for jet fuel applications (4). Beyond its role as a fuel, isobutanol has also been widely used as an industrial solvent and chemical feedstock (8). Isobutanol can be dehydrated to produce isobutene, which serves as an important platform molecule for synthesizing renewable fuels and a wide range of chemical products (8). Because of these advantages, the isobutanol market is expanding and is projected to reach USD 2.59 billion by 2030 (9). An additional advantage of isobutanol is that it can be produced as a byproduct of fermentation by the yeast *Saccharomyces cerevisiae* (10, 11). It is synthesized from pyruvate through both the valine biosynthetic and Ehrlich pathways. *S. cerevisiae* possesses several advantageous traits as a host for biofuel production, including ease of genetic manipulation, the ability to grow in low-pH environments, and resistance to phage infections (12). Moreover, *S. cerevisiae* has a long history in beer brewing, baking, and industrial ethanol production. In addition, because large-scale fermentation facilities are already in place, transitioning to isobutanol production is economically feasible with minimal modifications to existing equipment (12).

Nevertheless, the primary metabolic product of wild-type *S. cerevisiae* is ethanol, and its native capacity for isobutanol production remains extremely limited, thereby restricting its practical application (9). A major challenge is the low tolerance of *S. cerevisiae* to isobutanol, as high concentrations of isobutanol significantly inhibit its growth. Indeed, previous studies have reported that wild-type *S. cerevisiae* exhibits a growth reduction exceeding 50% when exposed to 1.4% isobutanol (13). To address this limitation, various metabolic engineering strategies, including pathway enhancement and the selection of tolerant strains, have been explored (14). However, effective strategies to improve isobutanol tolerance in *S. cerevisiae* have yet to be established, highlighting the need to elucidate the mechanisms underlying isobutanol-induced growth inhibition (15, 16). A key step toward this goal is identifying the protein targets of isobutanol. This task is inherently challenging because interactions between small molecules such as isobutanol and proteins are often transient, weak, or nonspecific. In particular, the concentration of isobutanol that inhibits growth (>1.4% w/v, approximately 190 mM) is much higher than the typical concentrations at which specific protein-ligand interactions occur, indicating that the binding affinity of isobutanol for its targets is likely to be low. These characteristics complicate enrichment, isolation, and detection of isobutanol-interacting proteins using standard biochemical methods such as affinity purification and mass spectrometry. Consequently, the specific protein targets of isobutanol in *S. cerevisiae* remain unidentified.

In our previous study, we demonstrated that isobutanol triggers a nitrogen starvation response in *S. cerevisiae via* the transcription factor Gln3p, even under nitrogen-rich conditions (13). This response leads to the upregulation of genes involved in amino acid biosynthesis and nitrogen utilization, whereas genes associated with glycolysis, cell wall biosynthesis, and membrane lipid biosynthesis are downregulated, thereby contributing to growth inhibition. Notably, deletion of *GLN3* improves yeast tolerance specifically to branched-chain alcohols, but not to linear alcohols (13). This branched-chain specificity suggests that the toxicity caused by branched-chain alcohols has a unique mechanism of action and that these alcohols induce specific cellular responses through a molecule that recognizes branched-chain structures in yeast.

In this study, we first found that the degree of isobutanol-induced growth inhibition in wild-type yeast is influenced by the leucine concentration in the medium. Based on this observation and our previous findings, we hypothesized that amino acids and their recognition mechanisms contribute to isobutanol-induced growth inhibition in yeast. To elucidate the mechanism underlying isobutanol-induced growth inhibition in yeast, we identified specific amino acids that mitigate isobutanol toxicity using tolerance tests under conditions of excess individual amino acids. We then investigated the interaction between isobutanol and yeast biomolecules that recognize these amino acids using saturation transfer difference nuclear magnetic resonance (STD-NMR) spectroscopy. Our results revealed that excess branched-chain amino acids (BCAAs), particularly leucine, alleviate yeast growth inhibition caused by isobutanol. Furthermore, we demonstrated that isobutanol acts as a competitive inhibitor of the interaction between leucine and leucyl-tRNA synthetase (LeuRS). These findings provide the basis for an atomic-level understanding of the mechanism by which isobutanol inhibits yeast cell growth and offer valuable insights for engineering yeast strains with enhanced tolerance to improve biofuel production.

## Results

### Effect of excess amino acids in culture medium on yeast isobutanol tolerance

**Figure 1:**
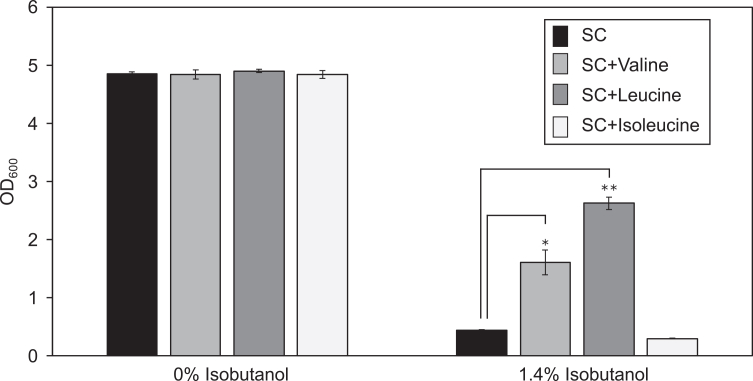
**Isobutanol tolerance of wild-type yeast in medium supplemented with excess BCAAs.** The effect of excess BCAAs on isobutanol-induced growth inhibition in wild-type yeast was assessed in SC medium containing 0% or 1.5% (v/v) isobutanol. Supplementation with excess branched-chain amino acids, particularly leucine, partially alleviated isobutanol-induced growth inhibition. Error bars represent the standard error of the mean (SEM) from three independent experiments. Statistical significance of the difference in cell growth between SC medium and medium supplemented with excess amino acids in the presence of isobutanol was determined using a two-tailed Student’s *t* test (∗*p* < 0.01, ∗∗*p* < 0.001).

To investigate the effects of amino acids in the culture medium on yeast isobutanol tolerance, we conducted isobutanol tolerance assays using media supplemented with excess amino acids. The addition of excess BCAAs significantly alleviated isobutanol toxicity but did not affect 1-butanol toxicity (Fig. S1). In contrast, the addition of excess tyrosine or other amino acids did not mitigate the toxicity of either alcohol. To identify which specific BCAA was responsible for the mitigation of isobutanol toxicity, we examined the effects of individual BCAAs. Excess leucine most effectively mitigated isobutanol toxicity (Fig. 1). A similar but weaker effect was observed with supplementation with excess valine, whereas isoleucine had no impact on toxicity mitigation. These results suggest that isobutanol acts on a biological mechanism involved in the recognition of BCAAs, particularly leucine.

### Ligand-binding analysis of LeuRS using STD-NMR

Aminoacyl-tRNA synthetases (aaRSs) catalyze the attachment of specific amino acids to their corresponding tRNAs, a process essential for protein biosynthesis (17, 18, 19, 20, 21). In this study, we focused on the initial step of the aminoacylation reaction, in which aaRSs specifically recognize and bind to their cognate amino acids. We hypothesized that isobutanol, owing to its structural similarity to leucine, interferes with the recognition of leucine by leucyl-tRNA synthetase (LeuRS). In particular, the isobutyl group of isobutanol closely resembles the side chain of leucine and shows greater structural similarity than that of valine, which contains an isopropyl group. In addition, leucine analogs such as leucinol, which have been reported to bind to LeuRS, share structural features with isobutanol (22). Based on these structural similarities, we investigated whether isobutanol could interact with LeuRS and potentially interfere with leucine recognition. To test this hypothesis, we analyzed the molecular interaction between LeuRS and isobutanol and evaluated how isobutanol affects leucine binding to LeuRS using STD-NMR spectroscopy. STD-NMR is a powerful technique for probing interactions between small molecules and their target proteins (23, 24). By selectively saturating the protein peaks and detecting magnetization transfer to bound ligands, this method enables identification of ligand protons involved in binding. Although STD-NMR does not yield precise dissociation constants, it provides a semi-quantitative estimation of interaction strength based on relative peak intensities when the ligands are of similar size, where higher STD intensities generally reflect stronger binding.

**Figure 2:**
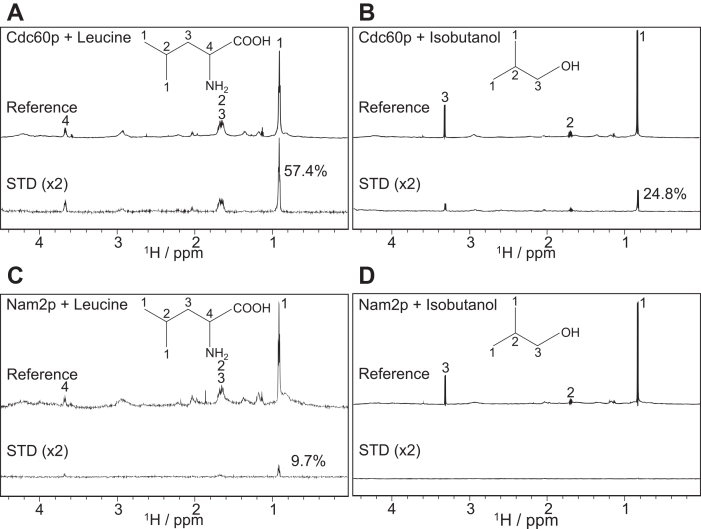
**STD-NMR analysis of interactions between LeuRS and ligands.** STD-NMR and reference spectra of Cdc60p in the presence of leucine (*A*) and isobutanol (*B*), and of Nam2p in the presence of leucine (*C*) and isobutanol (*D*). The peak labels correspond to the proton numbers indicated on the structural formula of each compound shown in the *panels*. The value at Peak 1 in the STD-NMR spectra were calculated as the ratio of each peak intensity to that of the corresponding peak in the reference spectrum. In *panel* (*D*), the relative intensity is below 2%. STD-NMR analyses demonstrated direct interaction of isobutanol with cytoplasmic LeuRS (Cdc60p), whereas interaction with mitochondrial LeuRS (Nam2p) was substantially weaker. Amide and hydroxyl proton peaks were not observed because the experiments were conducted in a D_2_O-based buffer.

STD-NMR analysis was performed using the cytoplasmic LeuRS (Cdc60p) and the mitochondrial LeuRS (Nam2p). These proteins were produced in *Escherichia coli*, purified without removing the thioredoxin tag, and verified to be enzymatically active (Fig. S2). STD-NMR spectra showed leucine peaks indicative of interactions with Cdc60p and Nam2p (Fig. 2, *A* and *C*). Control STD-NMR experiments using samples containing only protein or ligand suggested the absence of signals at the position corresponding to the leucine peak described above (Fig. S3, *A* and *B*). In addition, control experiments verified that thioredoxin, used as a tag for soluble protein expression, did not bind to either leucine or isobutanol (Fig. S4, *A* and *B*). These results indicate that the observed STD-NMR signals for leucine arise from specific interactions with Cdc60p or Nam2p. Among the observed STD-NMR peaks, the methyl group protons (Peak 1, 0.92 ppm) exhibited the highest intensity. This suggests that the methyl group in leucine interacts most strongly with Cdc60p and Nam2p, with relative intensities of 57.4% and 9.7% of the reference spectrum, respectively. The higher STD-NMR peak intensity observed for leucine binding to Cdc60p compared with Nam2p suggests that leucine binds more strongly to Cdc60p. These differences in binding affinity likely reflect structural variations in the leucine-binding pockets of the two LeuRS proteins. Since the methyl group (Peak 1) consistently showed the strongest interaction across all tested ligands, subsequent quantitative analyses focused on this peak. Similarly, STD-NMR analysis of Cdc60p with isobutanol revealed that the methyl group protons of isobutanol (Peak 1, 0.85 ppm) exhibited the highest peak intensity, indicating a strong interaction, with an intensity of 24.8% relative to the reference spectrum (Fig. 2*B*). In contrast, no interaction or only an extremely weak interaction was detected between Nam2p and isobutanol (Fig. 2*D*). These results suggest that isobutanol preferentially binds to cytoplasmic LeuRS rather than mitochondrial LeuRS, potentially inhibiting cytoplasmic Leu-tRNA synthesis.

To further investigate the ligand specificity of Cdc60p, which interacts with isobutanol, we examined its interactions with additional alcohols and BCAAs using STD-NMR. Cdc60p also interacted with 1-butanol, exhibiting a peak at 0.85 ppm with an intensity of 8.8% relative to the reference spectrum (Fig. S5*A*). In contrast, no interaction or only an extremely weak interaction was detected with ethanol, valine, or isoleucine (Fig. S5, *B*–*D*), as the observed STD-NMR peak intensity was below 2% of the reference spectrum. These results suggest that the interaction between ligands and Cdc60p is influenced by the presence and position of the methyl group in the ligands.

**Figure 3:**
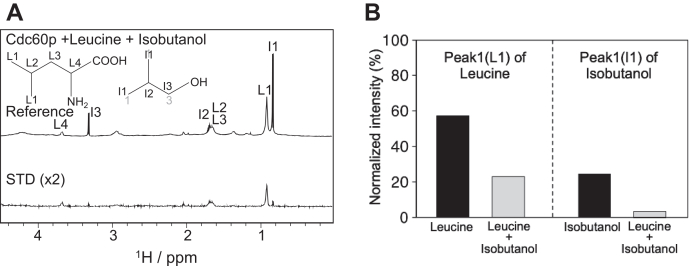
**Competition assay between leucine and isobutanol determined by STD-NMR.***A*, STD-NMR and reference spectra of Cdc60p in the presence of leucine and isobutanol. The peak labels correspond to the proton numbers indicated on the structural formulas of each compound shown in the *panel*. The values at Peak one in the STD-NMR spectra were calculated as the ratio of each peak intensity to that of the corresponding peak in the reference spectrum. *B*, the ratios of Peak 1 intensities in the STD-NMR spectrum to those in the corresponding reference spectra were compared between measurements of each ligand alone (*black*) and a mixture of leucine (L1) and isobutanol (I1). Competitive STD-NMR analyses suggested that isobutanol and leucine interact with overlapping binding regions in cytoplasmic LeuRS. In this experiment, ligand concentrations were adjusted so that the molar ratios of leucine to isobutanol was 1:1, as determined from the integrals of the reference peaks.

Next, to investigate whether leucine and isobutanol compete for binding to Cdc60p, STD-NMR experiments were performed using an equimolar mixture of the two ligands. Under these conditions, the STD-NMR peak intensities decreased for both ligands compared with the corresponding single-ligand experiments. The leucine signal decreased from 57.4% to 23.2% of the reference spectrum, corresponding to 40.4% of the intensity observed in the absence of isobutanol. Similarly, the isobutanol signal decreased from 24.8% to 3.5%, corresponding to 14.1% of the intensity observed in the absence of leucine (Fig. 3, *A* and *B*). The reduction in STD-NMR signal intensity for both ligands in the mixed condition suggests that leucine and isobutanol compete for overlapping binding regions on Cdc60p. The stronger residual signal observed for leucine further indicates that leucine binds to Cdc60p with higher affinity than isobutanol.

### Structure modeling of LeuRS

**Figure 4:**
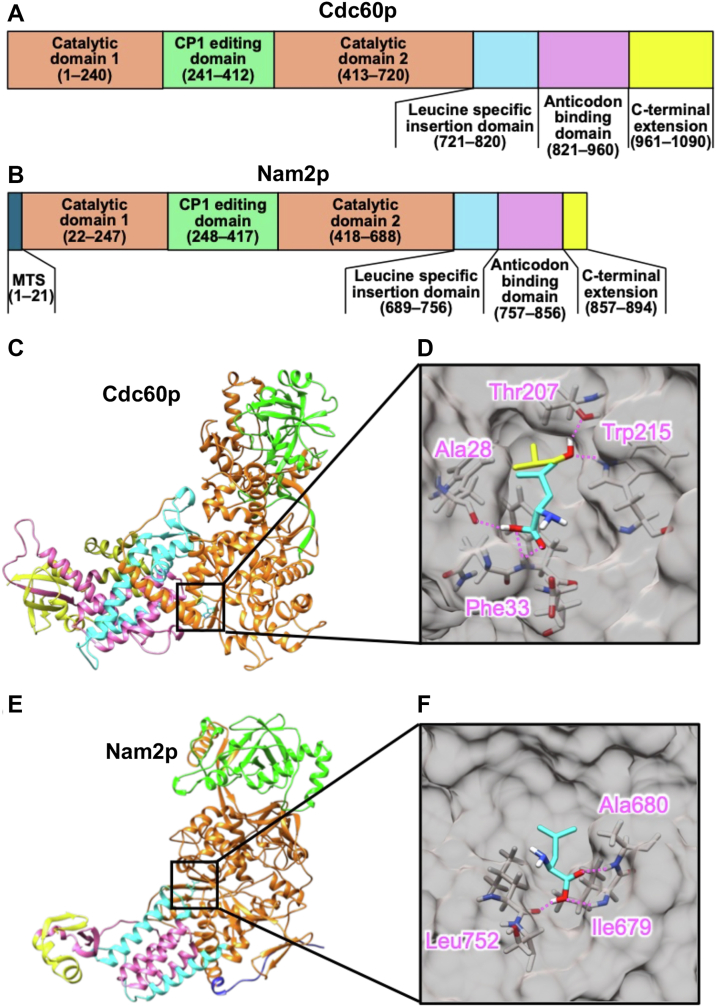
**Predicted structures of LeuRS complexes with leucine and isobutanol.** The amino acid numbers of each domain in Cdc60p (*A*) and Nam2p (*B*) are shown. Domain boundaries were assigned based on annotations from UniProt (Cdc60p: P26637, Nam2p: P11325), with numbers representing amino acid positions. Specific domains are colored: catalytic domain (*orange*), CP1 editing domain (*green*), leucine-specific insertion domain (*light blue*), anticodon-binding domain (*pink*), C-terminal extension domain (*yellow*), and MTS domain (*blue*). In the structural models of Cdc60p (*C*) and Nam2p (*E*), the colors of the structural surfaces correspond to the domain colors shown in (*A*) and (*B*). Leucine and isobutanol are depicted in *cyan* and *yellow*, respectively. Panel (*D*) shows the Cdc60p binding pocket with leucine and isobutanol overlaid, whereas panel (*F*) shows the Nam2p binding pocket containing only leucine. Docking analyses predicted favorable binding of isobutanol within the catalytic domain of Cdc60p but not within the corresponding pocket of Nam2p.

STD-NMR analyses revealed that leucine binds to both Cdc60p and Nam2p, whereas isobutanol interacts exclusively with Cdc60p, suggesting distinct binding pocket geometries between these proteins. Accordingly, we modelled the three-dimensional structures of Cdc60p and Nam2p using AlphaFold, which revealed overall structural differences in the amino acid residues forming their leucine-binding pockets (Fig. 4, *C* and *E*). We further explored these differences by performing docking simulations with AutoDock, modeling each ligand–protein pair: leucine–Cdc60p, isobutanol–Cdc60p, leucine–Nam2p, and isobutanol–Nam2p. LeuRS contains two functionally distinct substrate-binding sites: the synthetic (aminoacylation) site within the catalytic core, which activates leucine and transfers it to tRNA, and the CP1 editing site, which hydrolyzes mischarged tRNAs generated from near-cognate amino acids such as norvaline (25). Docking simulations using full-length structural models consistently identified energetically favorable docking poses for leucine and isobutanol within the catalytic aminoacylation pocket rather than within the CP1 editing site. These results further support the competitive binding observed in the STD-NMR experiments using Cdc60p, thereby reinforcing the conclusion that isobutanol and leucine share a common binding region within the aminoacylation active site. Notably, the leucine-binding pockets of Cdc60p and Nam2p are located within the catalytic (aminoacylation) domain (Fig. 4, *A* and *B*), which includes the KMSKS motif, consistent with previous reports identifying this region as the leucine-binding site. This observation supports the validity of the predicted binding pocket locations (25, 26). Docking simulations predicted that isobutanol is accommodated within the same catalytic aminoacylation pocket as leucine in Cdc60p. In contrast, the predicted binding site of isobutanol in Nam2p was located distant from the catalytic domain and did not overlap with regions implicated in aminoacylation. The STD-NMR result showed negligible binding between isobutanol and Nam2p, suggesting that this interaction was non-specific and unlikely to represent a physiologically relevant binding site.

Analysis of the Cdc60p–ligand complex revealed that the amino group of leucine forms a hydrogen bond with the amide NH of Phe33, while its carboxyl group forms a hydrogen bond with the carbonyl group of Ala28. The binding pocket exhibited a surface area of 134.7 Å^2^, with 50% composed of hydrophobic residues such as Phe, Ile, and Ala. The leucine side chain was located within 4 Å of these residues, indicating a significant contribution of hydrophobic interactions to ligand binding. Consistent with these observations, molecular docking using AutoDock Vina predicted a free energy change of −5.2 kcal/mol for the leucine–Cdc60p complex formation, indicating a relatively strong interaction. Isobutanol was also accommodated in the same pocket, forming hydrogen bonds between its hydroxyl group and both the indole nitrogen of Trp215 and the hydroxyl group of Thr207, with a predicted binding free energy change of −3.6 kcal/mol (Fig. 4*D*). For Nam2p, the amino group of leucine formed a hydrogen bond with the amide NH of Ala680, while the carboxyl group established hydrogen bonds with the carboxyl group of Leu752 and the amino group of Ile679. The binding pocket in Nam2p had a surface area of 85.4 Å^2^, which was 37% smaller than that of Cdc60p. Sixty percent of the pocket was composed of hydrophobic residues such as Leu, Ile, and Val, and the leucine side chain was located within 4 Å of these residues, supporting the presence of hydrophobic interactions. The predicted binding free energy change of the leucine–Nam2p complex formation was −3.4 kcal/mol, notably weaker than that of the leucine–Cdc60p complex, further highlighting the structural and energetic distinctions between these binding sites (Fig. 4*F*). Among these findings, the difference in binding pocket surface area between the models is notable, as it may contribute to the stronger interaction of leucine with Cdc60p observed in the STD-NMR experiments. However, further kinetic analyses are required to definitively determine whether this structural feature is associated with binding affinity. Furthermore, the observation that leucine and isobutanol bind to the same site on Cdc60p suggests competitive binding, which would account for the observed inhibition of yeast growth by isobutanol.

## Discussion

This study demonstrates that the supplementation of excess BCAAs, particularly leucine, alleviates isobutanol-induced growth inhibition in *S. cerevisiae*, likely because extracellular amino acid availability has been reported to influence intracellular amino acid pools in yeast (27). A slight recovery of growth inhibition was also observed under valine supplementation conditions. Because leucine and valine share common intermediates in the branched-chain amino acid biosynthetic pathway in yeast, valine supplementation may indirectly influence intracellular leucine utilization (11). However, this interpretation requires further confirmation because intracellular BCAA concentrations were not quantified in this study. Based on this observation, we further found that isobutanol competitively inhibits the binding of leucine to cytoplasmic LeuRS (Cdc60p). In contrast, although isobutanol is presumed to permeate the mitochondrial membrane similarly to other short-chain alcohols such as ethanol (14, 28, 29), STD-NMR and structural analyses revealed no detectable interaction between isobutanol and mitochondrial LeuRS (Nam2p). These findings indicate that the cytoplasmic isoform of LeuRS alone is involved in the isobutanol-induced growth inhibition observed in *S. cerevisiae*. The intracellular concentration of Cdc60p in *S. cerevisiae* has been estimated to be approximately 0.4 to 1 μM, whereas intracellular leucine levels under nutrient-rich conditions have been reported to range from one to two mM (17). Although intracellular amino acid levels can vary depending on the growth medium, these values provide an approximate order-of-magnitude comparison (30). Because small aliphatic alcohols like isobutanol easily cross the plasma membrane *via* passive diffusion, their intracellular concentrations rapidly equilibrate with the extracellular environment (31). Therefore, under growth-inhibitory conditions with 1% (v/v) isobutanol, the intracellular isobutanol concentration is estimated to reach approximately 108 mM (31).

Our STD-NMR analysis detected the binding of isobutanol to Cdc60p even at a relatively modest 20-fold molar excess. These results suggest that under physiological conditions (Cdc60p concentration is 1 μM and isobutanol concentration can reach 108 mM), the ligand-to-protein ratio far exceeds that of the experimental conditions. Thus, isobutanol can effectively compete with leucine for binding to LeuRS encoded by Cdc60p, thereby potentially inhibiting its aminoacylation function.

LeuRS is the aminoacyl-tRNA synthetase responsible for specifically recognizing leucine and catalyzing the formation of leucyl-tRNA (Leu-tRNA) (18). In all domains of life, aaRSs are essential enzymes that ensure translational fidelity and efficiency by catalyzing the initial step of protein synthesis—the aminoacylation of tRNAs with their cognate amino acids (19, 20, 21, 32, 33, 34, 35, 36). Additionally, Leu-tRNA functions as a key signaling molecule that modulates cell growth and metabolism through the target of rapamycin complex 1 (TORC1), a central regulator that couples amino acid availability to cell proliferation and biosynthesis (37, 38, 39, 40, 41, 42).

The competitive inhibition of the LeuRS–leucine interaction by isobutanol is expected to suppress Leu-tRNA synthesis, which would lead to the accumulation of uncharged tRNAs and activation of the general amino acid control (GAAC) pathway (43). Consequently, translation initiation is inhibited through the Gcn2-mediated phosphorylation of eIF2α (44). Furthermore, reduced Leu-tRNA synthesis is likely to impair Gtr1-mediated activation of TORC1, leading to TORC1 inactivation and subsequent suppression of protein synthesis and cell growth (45). Thus, isobutanol likely suppresses cellular translation by perturbing both aminoacylation and nutrient signaling pathways. To confirm this mechanism, directly quantifying the downstream inhibition of global protein translation *in vivo* remains an important objective for future research. Meanwhile, under high-concentration conditions, additional cytotoxic effects caused by membrane-permeable isobutanol may also contribute to growth suppression, and further studies are required to clarify the relative contributions of LeuRS inhibition and other cellular stress responses to isobutanol toxicity (14, 28, 29).

Structural predictions indicate that both leucine and isobutanol bind to the same pocket within Cdc60p. Although LeuRS possesses both aminoacylation and editing sites, the present docking simulations predicted preferential binding of isobutanol within the catalytic aminoacylation pocket rather than the CP1 editing domain. Leucine forms three hydrogen bonds, whereas isobutanol forms only two, consistent with the relative binding affinity observed in STD-NMR experiments. These results suggest that the number of hydrogen bonds contributes substantially to overall binding affinity. In addition to hydrogen bonding, both ligands engage in multiple hydrophobic interactions. Notably, both leucine and isobutanol interact with Phe33 *via* their methyl groups, suggesting that competitive binding might primarily occur at this site through hydrophobic interactions. Comparative analysis of ligand binding to Cdc60p and Nam2p further highlights structural and functional distinctions between the two enzymes. In Nam2p, leucine forms three hydrogen bonds, similar to its interaction with Cdc60p. However, the leucine-binding pocket of Nam2p is 37% smaller in surface area than that of Cdc60p, which likely limits the number of residues available for interaction and results in weaker binding of leucine to Nam2p. Such structural constraints may also account for the absence of detectable interaction between isobutanol and Nam2p. Taken together, these results suggest that the binding affinity and ligand specificity of Cdc60p and Nam2p are influenced not only by the number and nature of intermolecular interactions but also by the three-dimensional architecture of the binding pocket. These structural interpretations, however, are based on *in silico* modeling and therefore require further experimental validation.

In conclusion, this study demonstrates that isobutanol directly interacts with cytoplasmic LeuRS (Cdc60p) in yeast and competitively inhibits its binding to leucine. Our findings elucidate a molecular mechanism by which isobutanol impairs yeast cell growth and provide new insights into its cytotoxic mode of action. Importantly, understanding this mechanism could inform strategies to enhance yeast tolerance to isobutanol, thereby contributing to improved isobutanol production.

## Experimental procedures

### Yeast alcohol tolerance assay in the presence of excess amino acids

Alcohol tolerance assays were performed using the yeast *S. cerevisiae* strain BY4741 (Euroscarf) (46) in media supplemented with excess amino acids. The BY4741 strain was cultured in four types of liquid media: SC medium (Table S1), SC + BCAAs (supplemented with excess l-leucine, l-valine, and l-isoleucine), SC + Tyr (l-tyrosine), and SC + AAs (l-alanine, l-arginine, l-asparagine, l-glutamine, l-glycine, l-lysine, l-methionine, l-phenylalanine, l-proline, l-serine, l-threonine, and l-tryptophan). All amino acids were purchased from Nacalai Tesque, and added at twice their standard concentrations. The twofold supplementation level was based on a previous study (47). l-Histidine and l-cysteine were excluded because excess amounts have been reported to inhibit yeast growth (48), and l-tyrosine, which is less soluble than other amino acids, was not included in SC + AAs. Leucine was supplemented at a higher concentration than the other BCAAs, consistent with the standard composition of SC medium and in accordance with previous reports (49). Yeast cells were precultured in SC medium at 30 °C for 24 h. Then, the precultured cells were inoculated into each fresh medium containing either 1.4% (v/v) 1-butanol (Nacalai Tesque) or 1.4% (v/v) isobutanol (Nacalai Tesque) to achieve an initial optical density at 600 nm (OD_600_) of 0.1 in 96-well plates. The 96-well plates were sealed with aluminum foil tape (3M) and incubated at 30 °C for 24 h. After removing the tape, the OD_600_ value of each well was measured using a microplate reader (TECAN Infinite 200 PRO M Plex). To further investigate which specific BCAA mitigates the cytotoxicity of isobutanol, we prepared SC media supplemented with twice the standard concentration of l-valine (SC + Val), l-leucine (SC + Leu), or l-isoleucine (SC + Ile). Following the addition of 1.5% (v/v) isobutanol, yeast cells were cultured in 96-well plates, and cell growth was measured as described above.

### Plasmid construction for LeuRS expression

The genomic DNA of *S. cerevisiae* strain BY4741 was extracted using the Dr GenTLE High Recovery Kit (Takara Bio). The open reading frame (ORF) of the *CDC60* gene, which encodes the cytosolic leucyl-tRNA synthetase, and the ORF of the *NAM2* gene, which encodes the mitochondrial isoform, were amplified from the genomic DNA by one-step PCR as previously described (50). The primer sequences for *CDC60* amplification were 5′-ATGTTCTCTGGTTTGGTCTTAGAAAACACGG-3′ (*CDC60*-Fw) and 5′-TAAAATATTTTGGAAGACAACCTGGGTTACCTG-3′ (*CDC60*-Rv), and for *NAM2* amplification were 5′-ATGCTGTCTCGACCTTCAAG-3′ (*NAM2*-Fw) and 5′-TTACTTGTGGAATAAGAAAC-3′ (*NAM2*-Rv). The PCR products were inserted into the pET-32b vector (Merck KGaA) using TEDA seamless cloning (51). The resulting plasmids encoded the target proteins fused to an N-terminal His-tagged thioredoxin, facilitating affinity purification and improving protein solubility. The DNA sequences of the constructed plasmids (pET32b-*CDC60* and pET32b-*NAM2*) were verified by Sanger sequencing.

### Preparation of LeuRS proteins

Each plasmid was transformed into *E. coli* Rosetta (Sigma-Aldrich). Selected transformants were inoculated into 50 ml of LB medium containing 100 μg/ml ampicillin and 40 μg/ml chloramphenicol, and incubated at 37 °C for 12 h. The pre-culture was then transferred into 2 L of LB medium containing 100 μg/ml ampicillin and 40 μg/ml chloramphenicol, and the culture was grown at 37 °C. When the OD_600_ reached 0.7, the temperature was lowered to 15 °C, and protein expression was induced by adding 0.5 mM isopropyl β-D-1-thiogalactopyranoside (IPTG). After 16 h of incubation at 15 °C, the cells were harvested by centrifugation, and the resulting pellet was stored at −80 °C.

For purification of Cdc60p, cell pellets were thawed in lysis buffer containing 50 mM NaH_2_PO_4_ (pH 8.0), 300 mM NaCl, 10 mM imidazole, 20 mM β-mercaptoethanol (β-ME), 0.5 mM phenylmethylsulfonyl fluoride (PMSF), and 0.1 mM EDTA-free protease inhibitor cocktail (Nacalai Tesque) and sonicated on ice. The lysate was then centrifuged, and the resulting supernatant was loaded onto a HisTrap HP column using ÄKTA go (Cytiva), which was subsequently equilibrated with buffer containing 50 mM NaH_2_PO_4_ (pH 8.0), 300 mM NaCl, 10 mM imidazole, 20 mM β-ME, and 0.5 mM PMSF. The bound protein was then eluted with the same buffer by gradually increasing the imidazole concentration to 500 mM. The eluted protein-containing fractions were collected, and the NaCl concentration was diluted to 10 mM with 50 mM NaH_2_PO_4_ (pH 8.0). The diluted sample was then subjected to anion-exchange chromatography using a Q Sepharose High-Performance column (Cytiva) equilibrated with buffer containing 50 mM NaH_2_PO_4_ (pH 7.5), 10 mM NaCl, 20 mM β-ME, and 0.5 mM PMSF. The protein was eluted using the same buffer by increasing the NaCl concentration to 1 M. The protein-containing fractions were pooled and concentrated to 5 ml by centrifugation using a 10-kDa MWCO Amicon Ultra Centrifugal Filter (Merck). The concentrated sample was further purified *via* size-exclusion chromatography using a Superdex 200 column (Cytiva) and eluted with 50 mM PBS (pH 7.5) containing 20 mM β-ME and 0.5 mM PMSF. The molecular weight and purity of the purified protein were assessed by SDS-PAGE. Highly purified fractions were concentrated to 5 mg/ml and stored at 4 °C.

The purification of Nam2p was performed following the same procedure used for Cdc60p, except that cation-exchange chromatography was employed instead of anion-exchange chromatography. For cation-exchange chromatography, the protein sample was loaded onto an SP Sepharose High-Performance column (Cytiva) equilibrated with buffer containing 50 mM NaH_2_PO_4_ (pH 8.0), 10 mM NaCl, 20 mM β-ME, and 0.5 mM PMSF. The bound protein was eluted with the same buffer by increasing the NaCl concentration to 1 M. Although both LeuRS proteins were fused to a thioredoxin tag, attempts to remove the tag using enterokinase resulted in protein aggregation. Therefore, all analyses were conducted using LeuRS proteins retaining the thioredoxin tag.

### STD-NMR measurements

The purified Cdc60p solution was buffer exchanged into 50 mM PBS prepared in 99% D_2_O (pD 7.9) and concentrated to 30 μM. Each ligand, including individual BCAAs (leucine, valine, and isoleucine) and alcohol (isobutanol, 1-butanol, and ethanol; Kanto Chemical), was dissolved directly in the same buffer. NMR samples of 250 μl contained either 30 μM protein with 600 μM ligand, 30 μM protein alone, or 600 μM ligand alone. STD-NMR measurements were performed at 25 °C on an Avance II 700 MHz spectrometer equipped with a 5-mm ^15^N/^13^C/^1^H z-gradient triple resonance cryogenic probe (Bruker). The STD-NMR spectra were acquired using the pulse sequence reported previously (23). The radiofrequency (RF) irradiation was applied using a train of 50-ms Gaussian pulses with a flip angle of 720°, repeated for a total duration of 4 s. Two 1D spectra were acquired in interleaved mode with RF irradiation frequencies set to −1.0 and 2.5 ppm, with 128 scans per spectrum. The spectrum irradiated at −1.0 ppm corresponds to a region without NMR peaks from the protein, while 2.5 ppm corresponds to a region with protein peaks. The NMR data were processed using TopSpin (Bruker) with a line broadening of 0.3 Hz. The assignment of ligand NMR peaks was based on ^1^H NMR spectral data available in PubChem (52).

### Structure modeling

The structure models of Cdc60p and Nam2p, as well as their complexes with leucine or isobutanol, were constructed using UCSF Chimera (53) based on the structures obtained from the AlphaFold Protein Structure Database (for Cdc60p and Nam2p) and PubChem (51) (for leucine [CID 6106] and isobutanol [CID 6560]). These structures were energy-minimized using the MMFF94 force field in Chimera. For docking simulations, the binding sites of Cdc60p and Nam2p were defined according to previous studies (25, 26), and the docking grid was set to 20 × 20 × 20 Å (53). Docking of leucine and isobutanol to each protein was performed individually using the AutoDock Vina tool (54) implemented in Chimera with default parameters. Among the obtained docking results, the model with the most favorable predicted binding free energy (kcal mol^−1^) was selected, and analyzed for hydrogen-bond and hydrophobic interactions using Chimera’s built-in tools.

## Data availability

This article contains Supporting information. All data described in this study are contained within the manuscript and the supporting information.

## Supporting information

This article contains supporting information (55).

## Conflict of interest

The authors declare that they have no conflicts of interest with the contents of this article.
