# Supplementary material for: Leucyl-tRNA synthetase as a molecular target of isobutanol-mediated growth inhibition in Saccharomyces cerevisiae
Source: J Biol Chem. 2026 Jun 4;302(7):113228. doi: 10.1016/j.jbc.2026.113228 (PMC13355185; doi:10.1016/j.jbc.2026.113228)
Supplement: Supporting information [file mmc1.docx]

**Supporting information**

**Leucyl-tRNA Synthetase as a Molecular Target of Isobutanol-Mediated Growth Inhibition in *Saccharomyces cerevisiae***

Mano Hasegawa, Nodoka Oshimura, Kaho Hitomi, Ayako Furukawa, Kenji Sugase, Kouichi Kuroda

**Materials and methods**

**LeuRS enzymatic activity assay by ATP quantification**

The ATP Assay Kit–Luminescence (DOJINDO, Kumamoto, Japan) was used to evaluate the enzymatic activity of purified LeuRS isoforms, Cdc60p (a) and Nam2p (b) by quantifying ATP consumption during leucine binding. Reagent preparation followed a previously described method^1^, in which a reaction solution containing 5 µM ATP was supplemented with 10 µL of the working solution provided in the kit, followed by the addition of LeuRS at a final concentration of 0.1 µM. Leucine was subsequently added at final concentrations of 0, 1, 10, 100, 250, and 500 µM, and the mixtures were dispensed into white 96-well plates (NIPPON Genetics, Tokyo, Japan). The total reaction volume was 30 µL, and the mixtures were incubated at 30 °C for 10 min. To evaluate basal ATP hydrolysis in the absence of substrate, additional control reactions lacking leucine were performed using increasing concentrations of Cdc60p or Nam2p (0, 0.01, 0.025, 0.05, 0.1, 0.25, 0.5, and 1.0 µM) under otherwise identical conditions. Luminescence intensity was measured using an SH-9500Lab microplate reader (CORONA, Ibaraki, Japan) in luminescence mode with a detection wavelength of 556 nm, an integration time of 1 s per well, and an average of three measurements. The gain setting was set to automatic, and background correction was performed using wells without ATP as blanks. Luminescence intensity (RLU) was compared with an ATP standard curve to quantify ATP consumption. The ATP standard curve exhibited a strong linear relationship between luminescence intensity and ATP concentration (R² = 0.99), confirming the quantitative nature of this assay system.

**Reference**

[1] G. Enkhbat, A. Nakanishi and Y. Miki, The BRCA2 missense mutation K2497R suppressed self-degradation and increased ATP production and cell proliferation. *Biochem. Biophys. Res. Commun.* 590, 2022, 27–33.

**Table S1. Composition of synthetic complete (SC) liquid medium**

| **Components** | **Weight** | **Components** | **Weight** |
| --- | --- | --- | --- |
| Adenine | 95 mg | Isoleucine | 95 mg |
| *p-*Aminobenzoic acid | 9.5 mg | Leucine | 190 mg |
| Alanine | 95 mg | Lysine | 95 mg |
| Ammonium sulfate | 5.0 g | Methionine | 95 mg |
| Arginine | 95 mg | Phenylalanine | 95 mg |
| Asparagine | 95 mg | Proline | 95 mg |
| Aspartic acid | 95 mg | Serine | 95 mg |
| Cysteine | 95 mg | Threonine | 95 mg |
| Glutamic acid | 95 mg | Tryptophan | 95 mg |
| Glutamine | 95 mg | Tyrosine | 95 mg |
| Glycine | 95 mg | Uracil | 95 mg |
| Histidine | 95 mg | Valine | 95 mg |
| Inositol | 36 mg | Yeast nitrogen base without amino acids and ammonium sulfate | 1.5 g |
| Milli-Q water | | Up to 900 mL | |
| 20% (w/v) Glucose | | Add 100 mL after autoclaving | |

**Figure S1. Alcohol tolerance assay in amino acid excess medium.**

The effect of excess amino acids (BCAAs, Tyrosine, and AAs) in SC medium on yeast growth inhibition by alcohols was evaluated. Error bars represent the standard error of the mean (SEM) from three independent experiments. Statistical significance of differences in cell growth between SC medium with and without excess amino acids was determined using a two-tailed Student’s *t*-test (**p* < 0.05, ***p* < 0.01).

**Figure S2. LeuRS enzymatic activity assay by ATP quantification**

Under conditions of 0.1 µM Cdc60p and 500 µM leucine, ATP consumption was 2.11 ± 0.2 µM. The maximum ATP consumption (*C*_max_) was 2.2 ± 0.2 µM, and the Michaelis constant (*K*_m_) for leucine was 16.5 ± 0.2 µM (a). In contrast, under conditions of 0.1 µM Nam2p to 500 µM leucine, ATP consumption was 2.0 ± 0.2 µM. *C*_max_ was 2.0 ± 0.1 µM, and *K*_m_ for leucine was 18.2 ± 0.2 µM (b). When these values were compared with those under conditions without LeuRS, significant differences were observed (*p* < 0.05). These results indicate that purified LeuRS consumes ATP and binds to leucine, confirming its enzymatic activity. Control reactions lacking leucine showed negligible basal ATP hydrolysis (0.087 µM with 1 µM Cdc60p (c) and 0.084 µM with 1 µM Nam2p (d)). The data were obtained from three independent experiments (*n* = 3) and are presented as the mean ± standard deviation (SD). Similar experiments using isobutanol did not yield reliable measurements because alcohols caused large fluctuations in the luminescence signal of the ATP Assay Kit–Luminescence system.

**Figure S3. STD-NMR analysis under protein only conditions.**

STD-NMR spectra of Cdc60p only (a) and Nam2p only (b) conditions.

**Figure S4. STD-NMR analysis of interactions between thioredoxin and ligands.**

STD-NMR spectra of thioredoxin in the presence of leucine (a) and isobutanol (b). Peak labels correspond to proton numbers indicated on the structural formulas of each compound shown in the panels.

**Figure S5. STD-NMR analysis of interactions between yeast Cdc60p and various ligands.**

STD-NMR spectra of Cdc60p in the presence of 1-butanol (a), ethanol (b), valine (c), and isoleucine (d). Peak labels correspond to proton numbers indicated on the structural formulas of each compound shown in the panels. Values above Peak 1 in the STD-NMR spectra represent the relative intensity, calculated as the ratio of the peak intensity in the STD-NMR spectrum to that in the reference spectrum. Relative intensity values below 2% are not displayed.
